# Supplementary material for: Targeting DNA Damage Repair Mechanisms in Pancreas Cancer
Source: Cancers (Basel). 2021 Aug 24;13(17):4259. doi: 10.3390/cancers13174259 (PMC8428219; doi:10.3390/cancers13174259)
Supplement: Supplementary file 1 [file cancers-13-04259-s001.zip › cancers-1336277-supplementary.pdf]

Supplementary Material: “Targeting DNA Damage Repair Mechanisms in Pancreas Cancer”

Table S1: List of selected commercially available germline testing panels [6] referenced to the proposed ESDO expert panel of Table 2.

Ambry PancNext

"BRCA1" "BRCA2" "PALB2" "MLH1" "MSH2" "MSH6" "PMS2" "EPCAM" "ATM" "CDKN2A" "APC" "STK11" "TP53" "SPINK1" "PRSS1" "CFTR" "CTRC" "CASR"

Blueprint Genetics Hereditary Pancreatic Cancer Panel "BRCA1" "BRCA2" "PALB2" "MLH1" "MSH2" "MSH6" "PMS2" "EPCAM" "ATM" "CDKN2A" "APC" "STK11" "TP53" "BMPR1A" "BUB1B" "FANCC" "MEN1" "NF1" "SMAD4" "TSC1" "TSC2" "VHL"

Invitae Pancreatic Cancer Panel
"BRCA1" "BRCA2" "PALB2" "MLH1" "MSH2" "MSH6" "PMS2" "EPCAM" "ATM" "CDKN2A" "APC" "STK11" "TP53" "SPINK1" "PRSS1" "CFTR" "CTRC" "CASR" "BMPR1A" "SMAD4" "NF1" "MEN1" "VHL" "TSC1" "TSC2" "CDK4" "PALLD" "FANCC"

GeneDx Pancreatic Cancer Panel
"BRCA1" "BRCA2" "PALB2" "MLH1" "MSH2" "MSH6" "PMS2" "EPCAM" "ATM" "CDKN2A" "APC" "STK11" "TP53" "SPINK1" "PRSS1" "CFTR" "CTRC" "CASR" "VHL" "CDK4"

Fulgent Pancreatic Cancer Comprehensive Panel
"BRCA1" "BRCA2" "PALB2" "MLH1" "MSH2" "MSH6" "PMS2" "EPCAM" "ATM" "CDKN2A" "APC" "STK11" "TP53" "SPINK1" "PRSS1" "CFTR" "CTRC" "CASR" "BMPR1A" "SMAD4" "NF1" "MEN1" "VHL" "TSC1" "TSC2" "CDK4" "FANCC"

Myriad myRisk Hereditary Cancer
"BRCA1" "BRCA2" "PALB2" "MLH1" "MSH2" "MSH6" "PMS2" "EPCAM" "ATM" "CDKN2A" "APC" "STK11" "TP53" "CKD4" "BMPR1A" "SMAD4"

Color Hereditary Cancer Test "BRCA1" "BRCA2" "PALB2" "MLH1" "MSH2" "EPCAM" "ATM" "CDKN2A" "APC" "STK11" "TP53" "BMPR1A" "SMAD4"

Orange Overlapping with proposed panel by the ESDO expert committee
